# The customization of general outcome prediction models: a statistical exercise or a necessity?

**DOI:** 10.62675/2965-2774.20260461

**Published:** 2026-03-16

**Authors:** Rui Moreno, Maria del Pilar Arias López, Stefano Finazzi

**Affiliations:** 1 Unidade Local de Saúde de São José Hospital São José Lisboa Portugal Hospital São José, Unidade Local de Saúde de São José, Lisboa, Portugal.; 2 Nova Medical School Faculdade de Ciências Médicas de Lisboa Lisboa Portugal Faculdade de Ciências Médicas de Lisboa, Nova Medical School - Lisboa, Portugal.; 3 Universidade da Beira Interior Faculdade de Ciências da Saúde Covilhã Portugal Faculdade de Ciências da Saúde, Universidade da Beira Interior - Covilhã, Portugal.; 4 Hospital del Niños Ricardo Gutierrez Pediatric Intensive Care Unit Buenos Aires Argentina Pediatric Intensive Care Unit, Hospital del Niños Ricardo Gutierrez - Buenos Aires, Argentina.; 5 Sociedad Argentina de Terapia Intensiva Buenos Aires Argentina Sociedad Argentina de Terapia Intensiva - Buenos Aires, Argentina.; 6 Mario Negri Institute for Pharmacological Research IRCCS Department of Medical Epidemiology Laboratory of Clinical Data Science Lombardia Italy Laboratory of Clinical Data Science, Department of Medical Epidemiology, Mario Negri Institute for Pharmacological Research IRCCS - Lombardia, Italy.

Since 1985, with the publication of the second edition of the Acute Physiology and Chronic Health Evaluation II (APACHE II) score,^(1)^ predicting mortality in groups of adult patients admitted to intensive care units (ICUs) has become a daily practice. Shortly after, comparing actual with predicted mortality laid the foundation for calculating the Standardized Mortality Ratio (SMR) as the primary method for assessing ICU performance. Additionally, the ratio of expected mortality to resource use (SRU) became in 2007 one of the most reliable measures of an ICU's cost-effectiveness.^(2)^

At the same time, SMR and SRU are among the most common quality indicators used to benchmark and compare performance across national and regional registries,^(3)^ such as the Australian and New Zealand Intensive Care Society (ANZICS) Registry (which collects data on over 98% of all ICUs in Australia and nearly 70% in New Zealand), or the Intensive Care National Audit and Research Centre (ICNARC), which gathers and processes data on a significant number of British ICUs. However, one of the main weaknesses of the SMR and SRU depends on the accuracy of the Outcome Prediction Model (OPM) used to predict mortality. Several publications have long recognised that models’ calibration (the degree of correspondence between actual mortality in the target population and the OPM's estimated probability) can change over time and by region where the score is intended to be applied.^(4)^ Changes in management strategies, admission and discharge policies, case mix, and end-of-life decisions that occur over time typically lead to a gradual mismatch between the mortality predicted by the OPM and the actual mortality.^(5)^ To date, most major registries use a modified or customised version of the score based on data from the previous year to estimate mortality. This change in coefficients is usually not published, as is the case with the Intensive Care National Audit & Research Centre (ICNARC), the Australian and New Zealand Intensive Care Society (ANZIC, or the Austrian Centre for Documentation and Quality Assurance in Intensive Care (ASDI).

In Brazil and Uruguay, the "Organizational CHaractEriSTics in cRitical cAre" (ORCHESTRA) collaboration collects data across a large number of ICUs and issues yearly reports on their performance within a global benchmarking system. The registry uses the global equation of the original Simplified Acute Physiology Score (SAPS) 3 to calculate the SMR. In this setting, the overall equation of the third version of the SAPS (SAPS 3), published in 2005,^(6,7)^ has been shown to perform well, even more than 10 years after its publication,^(8)^ except in particular groups of patients, such as those with coronavirus disease 2019 (COVID-19).^(9)^ However, this should not prevent the responsible parties of these registries from regularly checking calibration (the difference between the predicted and the actual mortality) and discrimination (the model's capacity to distinguish between patients who died and those who survived), as well as the uniformity of fit (the performance of the OPM in different patient typologies, to detect subpopulations within the target population where the model does not perform well,^(10)^ since different populations can have very distinct characteristics even within the same pathological condition, what can lead to a difference performance of the OPM in different sub-groups).^(11)^ Any significant change in the OPM's performance must trigger recalibration before applying the OPM for benchmarking purposes, computing, and publishing the benchmarking data report.

This recalibration process can be performed using two main approaches.^(12)^ In first-level customisation, aimed at fixing calibration issues, a new logistic regression model is developed using the original coefficients and variables of the original OPM and computing a new logistic regression equation to estimate the dependent variable (outcome or use of resources); if the problem relies on discrimination (which is quite rare but possible), a second-level customisation approach is necessary, in which the coefficients for each variable are re-computed while not changing the variables, so that a new logistic regression equation can be developed to model the relationship between the original independent variables in the OPM and the observed outcome or use of resources (our dependent variable) in the global cohort. Then, the technique's success is tested in the global cohort by calculating the calibration and discrimination of the customised score on the database. This mandatory exercise is precisely the one that Soares et al. published in this issue of Critical Care Science.^(13)^ It is based on an extensive retrospective analysis of prospectively collected data from patients admitted to 177 ICUs (169 in Brazil and 8 in Uruguay) across 99 hospitals (91 in Brazil and 8 in Uruguay) between January 1st, 2022, and December 31st, 2023. After analysing the performance of the original global SAPS 3 equation, the authors found that the model overestimated mortality while maintaining good discrimination. This is not surprising, as most studies published in recent years have demonstrated a decrease in risk-adjusted mortality in ICUs.^(14,15)^ As recommended, the authors proceeded with a first-level customisation of SAPS 3 and validated it. Despite the use of the old Hosmer-Lemeshow C and H tests,^(16)^ which are very sensitive to the sample size - in this case, massive - instead of the more robust calibration belts^(17)^ and an incomplete evaluation of the uniformity of fit (which can only be partially deduced from the data), the recalibrated model performed adequately. Consequently, it should– until its performance deteriorates again – serve as the basis for the ORCHESTRA benchmarking program.

Congratulations to all participants and authors for publishing this exercise, as it provides an excellent demonstration of when and how recalibration can - and should—be effectively implemented.

## References

[1] Knaus WA, Draper EA, Wagner DP, Zimmerman JE (1985). APACHE II: a severity of disease classification system. Crit Care Med.

[2] Rothen HU, Stricker K, Einfalt J, Bauer P, Metnitz PG, Moreno RP (2007). Variability in outcome and resource use in intensive care units. Intensive Care Med.

[3] Jawad I, Rashan S, Sigera C, Salluh J, Dondorp AM, Haniffa R (2021). A scoping review of registry captured indicators for evaluating quality of critical care in ICU. J Intensive Care.

[4] Poncet A, Perneger TV, Merlani P, Capuzzo M, Combescure C (2017). Determinants of the calibration of SAPS II and SAPS 3 mortality scores in intensive care: a European multicenter study. Crit Care.

[5] Moralez GM, Amado FS, Martins GA, Nassar AP, Salluh JI (2025). How to use intensive care unit scoring systems: a practical guide for the intensivist. Crit Care Sci.

[6] Moreno RP, Metnitz PG, Almeida E, Jordan B, Bauer P, Campos RA (2005). SAPS 3 Investigators. SAPS 3—From evaluation of the patient to evaluation of the intensive care unit. Part 2: development of a prognostic model for hospital mortality at ICU admission. Intensive Care Med.

[7] Metnitz PG, Moreno RP, Almeida E, Jordan B, Bauer P, Campos RA (2005). SAPS 3 Investigators. SAPS 3—From evaluation of the patient to evaluation of the intensive care unit. Part 1: Objectives, methods and cohort description. Intensive Care Med.

[8] Moralez GM, Rabello LS, Lisboa TC, Lima MD, Hatum RM, De Marco FV (2017). ORCHESTRA Study Investigators. External validation of SAPS 3 and MPM_0_-III scores in 48,816 patients from 72 Brazilian ICUs. Ann Intensive Care.

[9] Kurtz P, Bastos LS, Ranzani OT, Soares M, Zampieri F, Hamacher S (2023). DP-EFFECT-BRAZIL investigators. Variants of concern and clinical outcomes in critically ill COVID-19 patients. Intensive Care Med.

[10] Moreno R, Apolone G, Miranda DR (1998). Evaluation of the uniformity of fit of general outcome prediction models. Intensive Care Med.

[11] Ranzani OT, Shankar-Hari M, Harrison DA, Rabello LS, Salluh JI, Rowan KM (2019). A Comparison of mortality from sepsis in Brazil and England: the impact of heterogeneity in general and sepsis-specific patient characteristics. Crit Care Med.

[12] Moreno R, Miranda DR, Fidler V, Van Schilfgaarde R (1998). Evaluation of two outcome prediction models on an independent database. Crit Care Med.

[13] Soares M, Borges LP, Burghi G, Kurtz P, Azevedo JR, Brandão CE, ORCHESTRA Study Investigators (2026). Contemporary validation of a SAPS 3 customized version in patients admitted to Brazilian and Uruguayan intensive care units: a multicenter cohort study. Crit Care Sci.

[14] Kaukonen KM, Bailey M, Suzuki S, Pilcher D, Bellomo R (2014). Mortality related to severe sepsis and septic shock among critically ill patients in Australia and New Zealand, 2000-2012. JAMA.

[15] Poole AP, Chaba A, Bellomo R, Bailey M, Deane A, Delaney A (2025). National Critical Care Research Platform Investigators (NCCR). Mortality trends for sepsis and septic shock among critically ill adults in Australia and New Zealand. Intensive Care Med.

[16] Hosmer DW, Lemeshow S, Klar J (1988). Goodness-of-fit testing for the logistic regression model when the estimated probabilities are small. Biom J.

[17] Finazzi S, Poole D, Luciani D, Cogo PE, Bertolini G (2011). Calibration belt for quality-of-care assessment based on dichotomous outcomes. PLoS One.

